# MECOPE: Multifocal excitation compressive-sensing photoacoustic endomicroscopy through a multimode fibre

**DOI:** 10.1016/j.pacs.2025.100733

**Published:** 2025-06-04

**Authors:** Tianrui Zhao, Edward Zhang, Paul C. Beard, Wenfeng Xia

**Affiliations:** a School of Biomedical Engineering and Imaging Sciences, King’s College London, 4th Floor, Lambeth Wing St Thomas’ Hospital, London SE1 7EH, United Kingdom; b Department of Medical Physics and Biomedical Engineering, University College London, Gower Street, London WC1E 6BT, United Kingdom; c Wellcome/EPSRC Centre for Interventional and Surgical Sciences, University College London, Charles Bell House, 67-73 Riding House Street, London W1W 7EJ, United Kingdom

**Keywords:** Photoacoustic imaging, Endoscopy, Multimode fibre, Compressive sensing, Wavefront shaping

## Abstract

Photoacoustic endoscopy has gained intensive research interest in recent years, particularly for guiding minimally invasive procedures in several clinical disciplines including oncology, cardiology and fetal medicine. Multimode fibres hold the potential to revolutionise medical endoscopy with ultrathin size and micrometre-level resolution. Compared to conventional endomicroscopes based on multi-core fibre bundles, multimode fibres-based endoscopes offer significantly higher spatial resolution, smaller diameters, and lower costs. However, current implementations of multimode fibre imaging, whether using raster-scan or speckle compressive sensing imaging, are hindered by limitations in frame rate or signal-to-noise ratio. In this work, we developed a multifocal excitation compressive-sensing photoacoustic endomicroscopy system that combines wavefront shaping-based light focusing with compressive sensing to achieve high imaging speed without compromising image quality. The method was validated through numerical simulations and experiments with carbon fibre phantoms and red blood cells *ex vivo*. Our results demonstrated comparable image quality to raster-scan-based imaging, while improving the frame rate by a factor of 5, reaching 11.5 frames per second. With further enhancements in focusing performance and the use of a higher repetition rate laser, this method shows promise for achieving real-time, high-resolution endomicroscopy through ultrathin probes, making it a valuable tool for guiding minimally invasive procedures.

## 1 Introduction

Photoacoustic imaging (PAI) is a hybrid modality that converts absorbed optical energy into acoustic signals, enabling the visualisation of optical absorption contrast at high spatial resolution and tissue penetration depth, providing detailed insights into tissue composition and functions [1], [2], [3], [4]. Building upon this foundation, photoacoustic endoscopy (PAE) emerged as a promising technique for in vivo imaging in clinical settings, particularly within internal organs and luminal structures [5], [6], [7], [8], [9]. PAE integrates the core principles of PAI into a miniaturised probe format, making it suitable for early stage cancer diagnosis and guiding minimally invasive procedures.

PAE probes have predominantly side-viewing configuration, optimised for imaging within tubular anatomical structures such as blood vessels and the gastrointestinal tract [6], [10], [11], [12], [13]. Recently, forward-viewing probes have attracted intense attention for guiding minimally invasive procedures such as tumour biopsies and fetal interventions, where accurate tissue characterisation directly ahead of the device is essential [14], [15], [16], [17]. Coherent fibre bundles (CFBs), commonly used in fibre-optic endoscopy, have been adapted for forward-viewing PAE. These systems support optical-resolution imaging through distal-end laser scanning [14], [15], [16], and have also been integrated with Fabry–Pérot sensors to enable all-optical photoacoustic computed tomography (PACT) [17]. Despite these advances, the lateral resolution remains limited, either by inter-core gaps within the fibre bundle in microscopy-mode imaging or by acoustically defined resolution, typically on the order of tens of micrometres. Higher resolution is essential for resolving finer tissue structures and enhancing diagnostic accuracy.

Recent advancements in wavefront shaping techniques have enabled the utilisation of multimode fibres (MMFs) as an alternative to CFBs for ultrathin endomicroscopy [18], [19], [20], [21], [22]. Through correction of light distortion during transport through MMFs via shaping the incident optical wavefront, it becomes feasible to generate and raster scan an optical focus at the distal MMF tip for microscopy imaging. Compared to CFBs, MMF-based endomicroscopy probes offer advantages such as higher pixel densities, smaller probe sizes, and reduced costs. MMF-based photoacoustic endomicroscopy was initially demonstrated using a piezoelectric transducer in a transmission-mode setup, though the bulky design limited its suitability for *in vivo* applications [23]. The incorporation of a fibre-optic ultrasound sensor enables significant miniaturisation of the probe. However, the use of liquid-crystal spatial light modulator limited the imaging speed to 30 s per frame due to the slow modulation rate [24]. To improve the imaging speed towards real-time imaging, more recent developments have integrated a fast digital micromirror device (DMD) for light wavefront shaping [25], [26]. High-fidelity photoacoustic endomicroscopy images of mouse red blood cells and ear vasculature *ex vivo* have been obtained at a spatial resolution of 1.2 μm. The highest scanning speed reached 47 kHz using a DMD subregion, supporting an imaging speed of 4.7 frames per second for the acquisition of images consisting of 10,000 pixels [25]. However, a higher rate around 20 Hz is highly desirable for applications requiring real-time imaging such as surgical guidance.

Compressive sensing has been recognised as a faster alternative to raster-scan-based approaches in MMF endoscopy [27], [28], [29]. In contrast to raster-scan based methods, which necessitate calibration of the MMF, compressive sensing-based approaches initially record a set of speckles at the distal fibre tip by altering the incident light field. For measurements, these speckles were used to illuminate the imaging object, while the excited fluorescence or photoacoustic signals were collected and converted into a signal vector. By making use of the sparsity as prior information, the images of objects could be reconstructed from the recorded speckles and the signal vector. The sparse nature of the imaging object allows for a smaller number of recorded speckle patterns than the number of pixels in the resulting image. Consequently, the rate of image acquisition could be enhanced by several times compared to raster-scan-based approaches, where the number of speckle patterns (in this case, optical focal patterns) equals the number of pixels. However, the use of random speckles for compressive sensing imaging through MMFs suffered from a low signal-to-noise ratio (SNR) in excited signals, leading to deterioration of imaging quality compared to raster-scan-based imaging on tissue samples [30]. Moreover, employing pre-recorded speckles as the measurement basis necessitates a high level of system stability. Environmental disturbances can induce decorrelation between pre-recorded speckles and those actually utilised in signal generation, resulting in a notable degradation in imaging performance. The combination of focused illumination and compressive sensing has been explored for super-resolution imaging, providing greater resilience to noise compared to speckle compressive sensing [31].

To cope with these challenges, we propose Multifocal Excitation Compressive-sensing Photoacoustic Endomicroscopy (MECOPE). This approach utilises wavefront shaping to produce multifocal excitation patterns for compressive sensing. Departing from conventional MMF-based compressive sensing methods, this approach replaces random speckles with multifocal excitation patterns, each containing multiple light foci. These patterns are generated using wavefront shaping through a real-valued intensity transmission matrix (RVITM) algorithm [26], [32]. The performance of the developed photoacoustic endomicroscopy probe was demonstrated with carbon fibres phantoms and mouse red blood cells. In comparison to raster-scan approach that measures a number of M spatial positions for the acquisition of an image comprising M pixels, multifocal excitation approach requires a smaller number of measurements before image reconstruction, facilitating higher imaging speeds compared to raster-scan imaging. Moreover, the adoption of multifocal excitation patterns as the measurement basis enhanced SNRs due to increased local light fluence, thereby improving image quality over speckle excitation compressive sensing methods. Additionally, simplifying the excitation pattern with multifocal excitation increased resilience to environmental vibrations compared to conventional speckle patterns, thereby preserving superior imaging performance in practical applications.

**Fig. 1:**
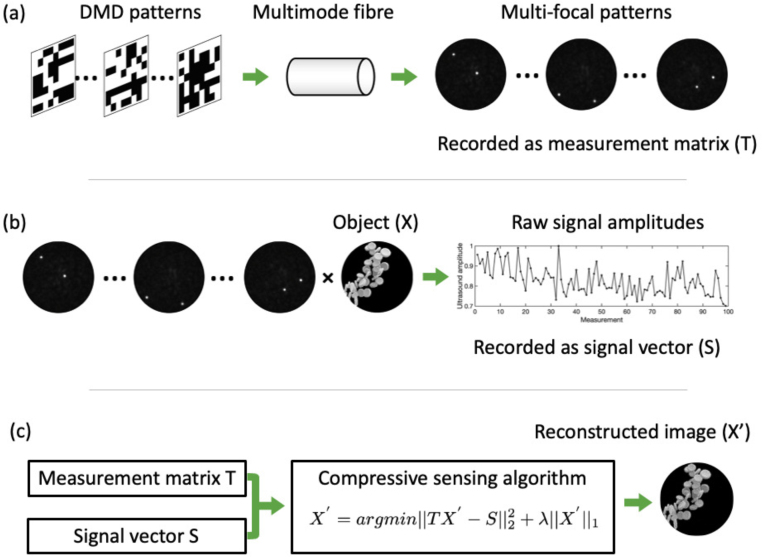
Principle of multifocal compressive sensing imaging. (a) Multifocal patterns acquisition. The multimode fibre were characterised with real-valued intensity transmission matrix to generate several foci (2 foci as an example) at multiple positions in each pattern by displaying optimal DMD patterns at the proximal fibre tip. These patterns were digitally recorded as the measurement matrix T. (b) Signal acquisition. The multifocal patterns were projected onto an object to generate signals. The amplitude of the raw signal generated by each multifocal pattern was recorded in order to form a signal vector. (c) Image reconstruction. The measurement matrix and the signal vector were fed into a compressive sensing algorithm to reconstruct the image of object.

**Fig. 2:**
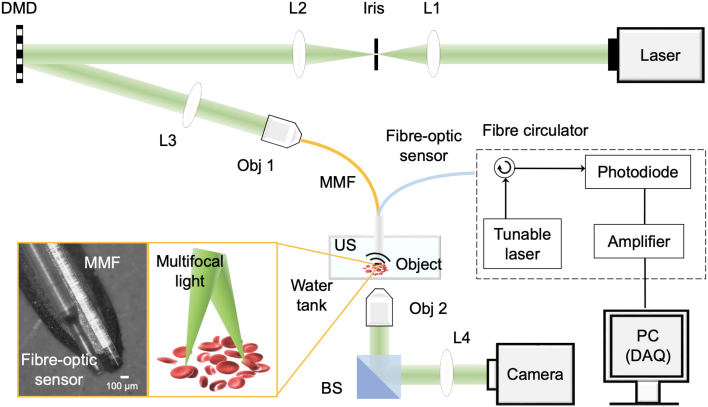
Illustration of the MECOPE system. Two inset images show the photo of the needle tip with a multimode fibre and a fibre-optic ultrasound sensor (left), and the schematic diagram of multifocal illumination on red blood cells. L1-4, convex lenses; DMD: digital micromirror device; US, ultrasound; Obj1-2: Objective lenses; MMF: multimode fibre; DAQ, data acquisition.

## 2 Materials and method

### 2.1 Multifocal excitation compressive sensing photoacoustic endomicroscopy

The principle of MECOPE imaging is illustrated in Fig. 1, which mainly comprises three major steps. In the first step, multifocal excitation patterns were generated via wavefront shaping (Fig. 1(a)). The RVITM algorithm, which was detailed in our previous studies [26], was used for single-point and multi-point focusing through a MMF. Briefly, a set of binary patterns were firstly projected to the proximal end of the MMF using a DMD, whilst the corresponding speckle patterns were captured at the distal end facet of the MMF with a CMOS camera. Subsequently, a RVITM was calculated to characterise the light transport through the MMF and to generate the optimal DMD patterns for light focusing at desired location of the MMF distal end facet. To focus light at multiple random positions, optimal DMD patterns corresponding to multiple random focusing positions were summed up and then converted into binary patterns by switching on micromirrors corresponding to a value larger than 0. By displaying these DMD patterns, a set of multifocal patterns were acquired as the measurement basis with each pattern converted into a row in the measurement matrix T.

In the second step, these patterns (measurement matrix T) were sequentially projected onto the object (X) to generate photoacoustic signals (signal vector (S), as shown in Fig. 1(b)). For each excitation pattern in T, the corresponding element in S represents the sum of the contributions from the generated photoacoustic signals from all the spatial locations in X, and can be expressed as:

(1) S=TX

In the third step (Fig. 2(c), the image of object $X^{\prime }$ was computationally reconstructed by solving an optimisation problem:

(2) $$X^{\prime }=argmin\|TX^{\prime }-S\|$$

With the pre-knowledge that the imaging target (natural objects) has a sparse representation and a non-negative intensity distribution in the reconstructed image, the equation used for image reconstruction in this case was re-written as:

(3) $$X^{\prime }=argmin{\left\|TX^{\prime }-S\right\|}_{2}^{2}+\lambda {\left\|X^{\prime }\right\|}_{1}$$

The second term, incorporating a L1-norm regularisation, which is commonly used to promote sparsity in the object. To achieve optimal results, the regularisation parameter λ should be carefully adjusted based on factors such as the SNR and the level of sparsity exhibited by the object. The computation in this study was achieved through the Fast Iterative Shrinkage-Thresholding Algorithm (FISTA) [33] for fast convergence rates.

### 2.2 Numerical simulations

Practical experiments are primarily affected by noise from environmental factors such as vibrations and variations in temperature which causes variations in the measurement patterns (fluctuation noise) as well as electronic readout noise from the detector (signal noise). Here we define the fluctuation noise as the difference between the intended light pattern and the actual pattern achieved through the fibre, which may arise from optical system vibration and random light noise. Given the difficulty of isolating these factors in experiments, we examined the resilience of speckle and multifocal excitation patterns to these noise sources through numerical simulations. First, we compared the imaging performance of random speckle and multifocal pattern measurement bases and investigated the impact of foci count on image reconstruction in MATLAB simulations under noise-free conditions. Both random speckles and multifocal patterns used in the simulations were captured experimentally. All measurement patterns have the same number of pixels (M = 100 × 100). Signal generation was modelled as the inner product of object pattern and each measurement pattern (1). The same number of speckles and patterns (K = 10,000) were collected and various numbers of illumination patterns were used for image reconstruction to investigate the impact of the measurement basis size.

To mimic natural fluctuation that leads to decorrelation between the pre-recorded illumination patterns and the ones used in signal generation, background noise was added to the experimentally recorded patterns to achieve varying decorrelation coefficients. The noise intensities at all patterns pixels followed a normal distribution. The fluctuation level, defined as the ratio of average of noise intensity to the mean intensity of the measurement patterns, was varied from 0 to 1.8 for both random speckles and multifocal patterns. Additionally, higher fluctuation levels, ranging from 2 to 3.6 times, were investigated for the 2-foci measurement basis. The fluctuated patterns were used to measure signals while the original patterns (before fluctuation) were used in image reconstruction through compressive sensing. To study the impact of signal noise, random noise ranging from 0.01 to 0.09 times the mean signal value was directly added to the signal vector. These noisy signals, along with the original measurement patterns, were used for image reconstruction. The quality of image reconstruction was evaluated using the Peak Signal-to-Noise Ratio (PSNR), a metric commonly employed in image compression, which is defined here via the mean squared error (MSE).

(4) $$MSE=\frac{1}{M}\overset{sqrt\left(M\right)-1}{\underset{i=0}{\sum }}\overset{sqrt\left(M\right)-1}{\underset{j=0}{\sum }}{\left[X\left(i,j\right)-X^{\prime }\left(i,j\right)\right]}^{2}$$

PSNR (in dB) is defined as;

(5) $$PSNR=20\log_{10}\left(MAX_{X}\right)-10\log_{\left(10\right)}MSE$$

where $MAX_{X}$ is the maximum pixel value of the object image.

**Fig. 3:**
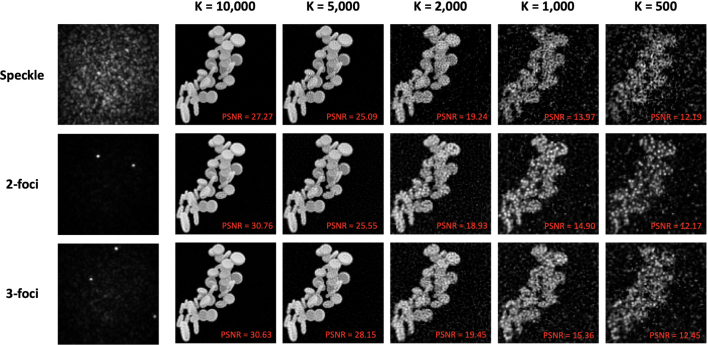
Numerical simulation of multifocal and random Speckle excitation compressive sensing imaging: Top row: Example of random speckle patterns and reconstructed images with varying measurement patterns (500 to 10,000). Middle and bottom rows: results of 2-foci and 3-foci measurements, respectively.

### 2.3 Experimental setup

The configuration of the optical and acoustic components integrated into the imaging system is illustrated in Fig. 2. A pulsed laser source (532 nm, 2 ns pulse duration; Elforlight, UK) provided the excitation light. High-speed spatial beam modulation was achieved using a digital micromirror device (DMD; 768 × 1080 pixels, DLP7000, Texas Instruments), where a region of 128 × 128 mirrors was selectively addressed to produce tailored illumination patterns. These patterns were relayed through an optics system consisting of a 50 mm focal length achromatic lens (Thorlabs, AC254-050-A-ML) and a 20× objective lens (NA = 0.4, Thorlabs, RMS20×), and then coupled into the proximal end of a 30 cm graded-index MMF (core diameter = 100μm, NA = 0.29; Newport, USA). For calibration purposes, the output pattern at the distal tip of the fibre was visualised using a second 20× objective in combination with a 100 mm focal length lens (Thorlabs, AC254-0100-A-ML), and with the image recorded by a CMOS camera (Hamamatsu C11440-22CU01).

For photoacoustic endomicroscopy imaging, the MMF was positioned alongside a fibre-optic ultrasound detector, with both fibres integrated into the lumen of a 22-gauge medical needle to form a compact, forward-imaging probe. The ultrasound sensor was based on a plano-concave Fabry–Pérot microresonator, which utilised a curved epoxy spacer enclosed between two reflective coatings. When exposed to acoustic waves, the cavity’s reflectivity changed due to deformation of the spacer material. A wavelength-tuneable continuous-wave interrogation laser (TSL-550, Santec, UK) was directed into the cavity using a fibre-optic circulator (Thorlabs, 1525–1610 nm range), and the back-reflected light was detected by a photodiode (Hamamatsu G9801-22). This signal was passed through a low-noise amplifier (SPA.1411, Spectrum Instrumentation) and digitised by a high-speed data acquisition board (M4i.4420, Spectrum Instrumentation). Data processing was performed on a desktop workstation equipped with an Intel i7 processor (3.2 GHz). Compared to conventional piezoelectric receivers, the all-optical ultrasound detector provided several advantages, including an ultracompact footprint (125μm diameter), near-omnidirectional sensitivity, and a broad detection bandwidth [34].

Following system calibration, the CMOS camera was removed and the needle tip was submerged into a custom-designed water tank to enable acoustic coupling between the imaging target and the fibre-optic ultrasound detector. Photoacoustic signals were captured by the fibre-optic sensor. System timing—including DMD control, laser triggering, and data acquisition—was coordinated using a programmable waveform generator (33600 A, Keysight, USA) and managed through a bespoke Matlab interface.

**Fig. 4:**
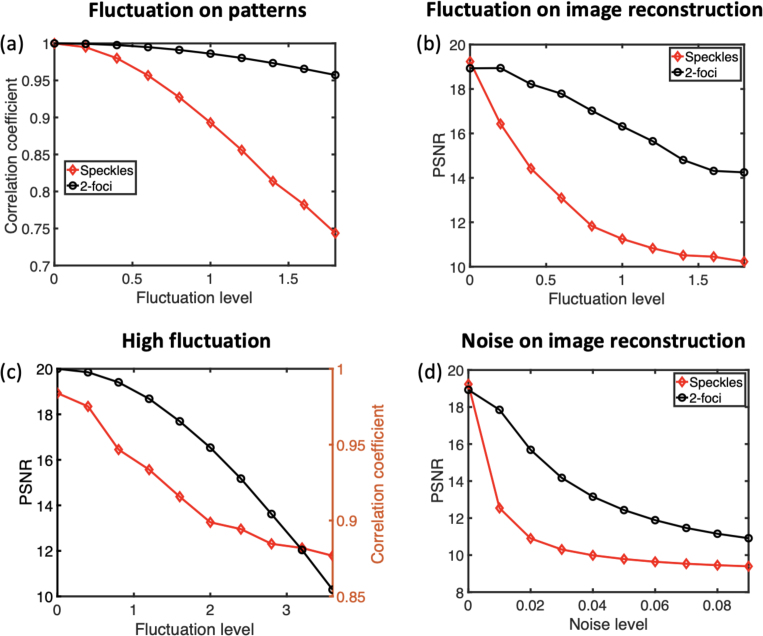
Impacts of non-ideal environment on compressive sensing imaging performance. (a) The evolution of correlation between the original pattern and the pattern after vibration with varying fluctuation level. (b) The evolution of imaging performance of 2-foci patterns and random speckles with varying fluctuation level. (c) The evolution of correlation and imaging performance of 2-foci compressive sensing imaging with high fluctuation level. (d) The evolution of imaging performance of 2-foci and random pattern compressive sensing imaging with varying noise level.

## 3 Results

### 3.1 Numerical simulation

A comparison between compressive sensing imaging using random speckles and multifocal measurement bases on the same object is presented in Fig. 3. Under ideal conditions, without any fluctuation or signal noise, images obtained with 2-foci and 3-foci bases demonstrated nearly the same image fidelity as those obtained with the random speckle basis. All types of measurement patterns allowed the number of measurements to be reduced to 5% of the total number of pixels in a single image, though this reduction resulted in a decrease in image quality, with PSNR dropping from approximately 30 to 12.

A total number of 2,000 measurement patterns were used to study the impact of fluctuation in patterns and noise in signals. We firstly investigated the resistance of patterns themselves to fluctuation noise. Since 2-foci and 3-foci patterns led to approximately the same results, only 2-foci results were used for comparison with random speckles. As shown in Fig. 4 (a), the 2-foci measurement patterns exhibited superior robustness to random fluctuations compared to speckle patterns, as evidenced by higher correlation coefficients between the perturbed and original patterns under equivalent fluctuation levels. This suggests that fluctuation noise has a smaller impact on multifocal compressive sensing imaging. This conclusion is supported by the results, which show that the 2-foci basis achieved a higher PSNR than the random speckle basis under conditions of fluctuation noise (Fig. 4(b)). While the PSNR of random speckle imaging dropped to ∼10 with a fluctuation level at 1.8, the PSNR achieved through 2-foci imaging was ∼14, the PSNR achieved with 2-foci imaging remained around 14, preserving the general structure of the object image as shown as results with K = 1,000 in Fig. 3. With continuous increase of the fluctuation level from 2 to 3.6 on 2-foci measurement patterns, the PSNR degraded to 10, whereas the correlation coefficients between original and fluctuated patterns declined to 0.87 (Fig. 4(c)). In addition to its higher resistance to fluctuation, the 2-foci basis also demonstrated greater resilience to random noise from signal detection. As indicated in Fig. 4 (d), it achieved higher PSNRs than the speckle basis at the same noise levels.

**Fig. 5:**
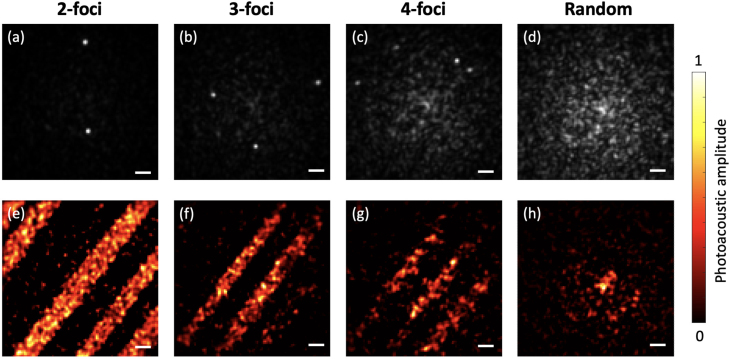
Photoacoustic imaging of the same object comprising carbon fibres. (a-d) Multifocal excitation patterns and random speckle. (e-h) Reconstructed images of a carbon fibre network with corresponding patterns. Scale: 5μm.

### 3.2 Photoacoustic imaging

Photoacoustic endomicroscopy imaging through a MMF with multifocal excitation compressive sensing was firstly demonstrated with carbon fibres. The increase in the number of foci does not affect the focus profile, as it is solely determined by the fibre core configuration. Each image comprised 10,000 pixels, covering an area of 50μm by 50μm. A total number of 2,000 patterns were used for the measurement of ultrasound signals. With the DMD running at 23 kHz, the rate of imaging acquisition reached 11.5 fps, which was 5 times faster than the raster-scan-based approach. The imaging performance of random speckle and multifocal pattern measurement bases was evaluated, with a comparison of the effects of varying the number of foci on image reconstruction. As shown in Fig. 5 (e-h), multifocal excitation patterns achieved higher imaging performance compared to random speckles. Using the same wavefront shaping approach and the same fibre, the laser focus maintains the same profile as demonstrated in our previous work [26], leading to the same lateral resolution. The focusing performance, evaluated by the enhancement factor, gradually declined with an increasing number of foci, and consequently, the image quality also degraded with more foci in each pattern. As illustrated in Fig. 5(h), speckle compressive sensing reconstructed the general structure of the carbon fibres only in the central region, where the highest light intensity was distributed at the tip of the graded-index (GRIN) MMF.

**Fig. 6:**
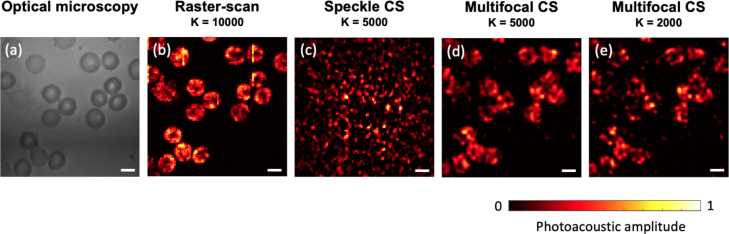
Photoacoustic imaging of mouse red blood cells. (a) Optical microscopy image (transmission-mode) of a mouse blood smear on a cover slip. (b) Photoacoustic image of the same sample with raster-scan mode. (c) Speckle compressive sensing with 5000 measurements. (d) Multifocal compressive sensing with 5000 measurements and 2000 measurements (e). Scale: 5μm.

The performance of the MECOPE was further demonstrated with biological tissue by using 2-foci patterns to illuminate a mouse blood smear sample on a coverslip. Mouse blood was obtained from culled mice. The procedures involving mice were ethically reviewed and carried out in accordance with the Animals (Scientific Procedures) Act 1986 (ASPA) UK Home Office regulations governing animal experimentation. The performance of MECOPE was compared with raster-scan photoacoustic endomicroscopy imaging and random speckle compressive sensing imaging in Fig. 6. The biconcave structures of red blood cells were visualised in raster-scan and 2-foci MECOPE, while random speckle compressive sensing failed to visualise the structure of red blood cells. As shown in Fig. 6 (d-e), 2-foci MECOPE enabled imaging with reduced numbers of measurements by factors of 2 and 5, resulting in frame rates of 4.6 and 11.5 frames per second (fps), respectively, compared to the original frame rate of 2.3 fps. The laser power was the same as the one used in our previous study [26]; the total energy at the single optical focus (1.2μm in diameter) was measured as ∼20 nJ which was 8.9% of the total output of the MMF, leading to a maximum optical fluence of 1.7 J/cm${}^{2}$ at the focal area in a raster-scan pattern. The total laser power provided by the laser machine was kept the same for all imaging modes. The enhancement factor of light focusing in 2-foci pattern was measured to be 0.2 times of the single focus, leading to an optical fluence of 0.34 J/cm${}^{2}$ at each focus.

## 4 Discussion

A compressive sensing method using multifocal excitation patterns has been demonstrated for MMF endomicroscopy imaging in both numerical simulations and experiments. With this approach, a miniature photoacoustic endomicroscopy imaging probe was developed using a highly sensitive and broadband fibre-optic ultrasound receiver. The performance of the imaging system was validated on a carbon fibre phantom and red blood cells *ex vivo*. Since the lateral resolution is determined by the laser focus profile and the axial resolution depends on the frequency and bandwidth of the fibre-optic ultrasound sensor, both resolutions and imaging depth are consistent with those achieved in our previous raster-scan-based imaging studies [25], [26]. Compared to raster-scan-based fibre-optic photoacoustic endomicroscopy imaging system from our previous studies [26], the acquisition speed for an image comprising 10,000 pixels has improved by up to 5 times, reaching 11.5 fps. To the best of our knowledge, this represents the fastest imaging speed achieved for photoacoustic endomicroscopy of biological tissue. Compared to previously reported photoacoustic compressive sensing imaging of red blood cells *ex vivo* that required 100 times signal averages, MECOPE allowed single-shot signal acquisition for each multifocal pattern, significantly reducing the acquisition time. Additionally, several seconds are needed to acquire the illumination patterns, depending on the number of patterns used. The acquisition speed is currently limited by the camera frame rate, while the DMD operates at 23 kHz and is not the bottleneck. It is worth noting that the computational cost could be further reduced by implementing GPU acceleration or optimised parallel processing strategies. With appropriate hardware improvements, near real-time RVITM acquisition and image reconstruction could be achievable, further enhancing the system’s practical applicability for dynamic imaging scenarios.

With the same image reconstruction algorithm, multifocal speckles with varying numbers of foci and random speckles exhibited nearly identical imaging performance in noise-free simulations. However, under experimental conditions where system vibrations and signal readout noise are unavoidable, multifocal patterns enabled the reconstruction of phantom structures, whereas increasing the number of foci led to a decline in image quality, and random speckles resulted in the lowest reconstruction performance. This degradation is likely due to the reduced light power density at focal regions, which lowers the SNR of the photoacoustic signals. This observation aligns with the finding that carbon fibre images reconstructed using random speckles primarily captured the central region, where light intensity is highest, while peripheral regions at the tip of a GRIN MMF were less resolved. Consequently, raster-scan imaging, which concentrates the highest local light power at the focal region, achieved the best image fidelity for red blood cell imaging, though it required five times more acquisition time compared to MECOPE. Averaging is effective for reducing electronic readout noise from the detector (signal noise), and it is also reasonable to expect that averaging can suppress variations in the measurement patterns (fluctuation noise), as supported by previous studies using iterative optimisation for light focusing through dynamic media [35].

In this work, we have demonstrated the feasibility of enhancing the imaging speed of a fibre-optic photoacoustic endomicroscopy system using compressive sensing, marking a significant step towards real-time imaging and clinical translation. This advancement holds strong potential for guiding minimally invasive procedures such as tumour biopsy. However, the current system operates at a single wavelength (532 nm), which primarily provides haemoglobin contrast. Future work should explore the full potential of multispectral and functional photoacoustic imaging, enabling the visualisation of a broader range of biomarkers, including haemoglobin, lipids, and nucleic acids, as well as functional parameters such as blood oxygenation, flow dynamics, and metabolic activity. By addressing these challenges, MECOPE could evolve into a powerful intraoperative tool capable of high-precision, *in situ* tumour assessment. This would ensure accurate, first-attempt targeting during biopsies, offering transformative benefits across a broad spectrum of cancers by: (1) improving tumour grading accuracy through reliable sampling of the most representative and aggressive regions; (2) minimising the risk of complications (e.g., haemorrhage, infection) by reducing the number of needle passes; and (3) reducing operating room time and healthcare costs by streamlining procedures.

While the system has been demonstrated on *ex vivo* cells, further improvements are needed to advance it towards practical use in clinical settings. It is suggested that concentrating more light energy into focal spots, which improves SNR of the ultrasound signal, has the potential to further improve the imaging performance of MECOPE. The multifocal patterns in this work were achieved via wavefront shaping using a DMD modulating the incident light wavefront. Since the DMD was used for binary amplitude modulations, the theoretical enhancement of local light intensity is N/2π, where N is the number of independently controlled micromirrors. As such, a larger number of micromirrors can be employed to enhance light focusing but with an increased characterisation time. DMDs have also been demonstrated for phase modulation based on the Lee hologram [36] and super-pixel [37] methods, with which the theoretical enhancement can be increased to πN/4. However, this method suffers from low energy efficiency, which poses a challenge for photoacoustic imaging that requires high light fluence for effective ultrasound generation. In this work, light fluence is primarily limited by the power of the laser source. Therefore, a laser with a higher repetition rate and greater pulse energy is highly desirable for achieving high-quality, high-speed photoacoustic endomicroscopy imaging through an MMF. Furthermore, with the same wavefront shaping approach and the same fibre, the variations on the profile of the laser focus are neglectable as shown in our previous work Ref. [26]. However, the laser output energy can fluctuate by up to 5%, which may lead to corresponding variations in the energy delivered at the foci. Compensating for these energy fluctuations could improve overall image quality, particularly in terms of uniformity. To address this, we plan to incorporate a photodiode in future work to monitor pulse-to-pulse energy variations.

Similar to single-point light focusing through a MMF, MECOPE relies on pre-characterisation of the MMF. Our previous studies have shown that minor bending can maintain focus quality and imaging performance, whereas large bending angles result in substantial degradation of focusing performance and reduce image fidelity [25], [26]. To address this, we used a graded-index (GRIN) MMF, which is known to better preserve coherent light transmission under moderate geometric perturbations [36]. To further enhance robustness against bending and enable practical deployment in realistic endoscopic scenarios, emerging strategies such as twisted core bundles [38] and spatial-frequency tracking adaptive beacons [39] could be integrated with MECOPE in the future, potentially achieving a fully flexible, high-speed photoacoustic endomicroscopy platform.

The field of view of 50μm x 50μm can provide sub-cellular visualisation of tissue immediately in front of the needle tip, supporting minimally invasive procedures such as needle biopsy. This capability is particularly valuable for improving tumour grading accuracy by facilitating reliable sampling of the most representative and aggressive tumour regions. The field of view can be increased by employing fibres with larger core diameters, as the image reconstruction algorithm can accommodate a greater number of pixels. One of the key advantages of using a MMF over a CFB in endomicroscopy is the ability to predefine focal depths during the system characterisation process. This allows both the field of view and spatial resolution to be scalable: as the imaging depth increases, the field of view expands, albeit at the expense of spatial resolution, as demonstrated in our previous work [40]. Additionally, the imaging depth is highly dependent on the wavelength of light used and the specific tissue chromophores targeted. A meaningful assessment of imaging depth will require in vivo studies, such as in an animal model where the needle probe is inserted directly into the tumour region, which is not feasible in the current study due to the bending induced image degradation. This will be another key focus of future work aimed at advancing the clinical translation of the technology.

## 5 Conclusion

In conclusion, we have developed a multifocal excitation compressive sensing method for photoacoustic endomicroscopy imaging using a MMF. This method significantly enhances imaging speed compared to traditional raster-scan imaging, while maintaining comparable image quality. The improved speed and efficiency of this approach hold great promise for advancing the development of real-time photoacoustic endomicroscopy probes, which could be useful for guiding various minimally invasive procedures with greater precision and effectiveness.

## CRediT authorship contribution statement

**Tianrui Zhao:** Writing – original draft, Visualization, Validation, Software, Methodology, Investigation, Formal analysis, Data curation, Conceptualization. **Edward Zhang:** Writing – review & editing, Software, Resources. **Paul C. Beard:** Writing – review & editing, Software, Resources. **Wenfeng Xia:** Writing – review & editing, Supervision, Project administration, Funding acquisition, Formal analysis.

## Declaration of competing interest

The authors declare the following financial interests/personal relationships which may be considered as potential competing interests: Wenfeng Xia, Tianrui Zhao has patent #UK Patent Application No. 2402014.1 pending to Wenfeng Xia, Tianrui Zhao. If there are other authors, they declare that they have no known competing financial interests or personal relationships that could have appeared to influence the work reported in this paper.

## Data Availability

Data will be made available on request.
